# Political Economy of Non-Communicable Disease (NCD) prevention and control in Lebanon: identifying challenges and opportunities for policy change and care provision reforms

**DOI:** 10.1186/s12889-023-17357-1

**Published:** 2023-12-18

**Authors:** Ibrahim R. Bou-Orm, Giulia Loffreda, Karin Diaconu, Sophie Witter, Pol deVos

**Affiliations:** 1 NIHR Global Health Research Unit on Health in Situations of Fragility, Institute for Global Health and Development, Queen Margaret University, Edinburgh, EH21 6UU UK; 2 Higher Institute of Public Health, Faculty of Medicine, Saint Joseph University of Beirut, Beirut, Lebanon

**Keywords:** Health policy, Non-communicable diseases, Health system reforms, Political economy analysis

## Abstract

**Introduction:**

Lebanon is a middle-income country facing substantial fragility features. Its health profile shows a high burden of NCD morbidity and mortality. This paper intends to analyse the political economy of NCD prevention and control in Lebanon.

**Methods:**

This study adopted a literature-based case study research design using a problem-driven political economy analysis framework. A total of 94 peer-reviewed articles and documents from the grey literature published before June 2019 were retrieved and analysed.

**Results:**

Lebanon’s political instability and fragile governance negatively affect its capacity to adapt a Health-in-All-Policies approach to NCD prevention and enable the blocking of NCD prevention policies by opposed stakeholders. Recent economic crises limit the fiscal capacity to address health financing issues and resulting health inequities. NCD care provision is twisted by powerful stakeholders towards a hospital-centred model with a powerful private sector. Stakeholders like the MOPH, UN agencies, and NGOs have been pushing towards changing the existing care model towards a primary care model. An incremental reform has been adopted to strengthen a network of primary care centres, support them with health technologies and improve the quality of primary care services. Nevertheless, outpatient services that are covered by other public funds remain specialist-led without much institutional regulation.

**Conclusion:**

Our study revealed a locked equilibrium in NCD prevention policymaking in Lebanon, but with an incremental progress in service delivery reforms towards a primary care model. Advocacy and close monitoring by policy entrepreneurs (such as civil society) could initiate and sustain the implementation of policy change and care model reforms.

**Supplementary Information:**

The online version contains supplementary material available at 10.1186/s12889-023-17357-1.

## Introduction

Despite the achievements in tackling Noncommunicable Diseases (NCDs) in high-income settings during the last century [1], global advocacy and debate around the NCD burden in low- and middle-income countries (LMICs) has only recently gained traction. This is due to several factors, including competing priorities, such as infectious diseases, lack of sustained advocacy and financing in this area, and the inherently complex nature of the multisectoral interventions to tackle NCDs. From a political perspective, NCDs were discussed as a global priority in three UN General Assembly meetings in 2011, 2014 and 2018, with a call for political commitment globally and catalyzing action against NCDs. Moreover, a global strategy and a global action plan for 2013 – 2020 were published to guide global efforts towards NCD prevention and control [2]. Cost-effective interventions emerged, including population-level approaches to NCD prevention tackling some ‘commercial determinants of health’ [3], such as tobacco and alcohol, as well as service delivery reforms to improve NCD management [2]. The second set of interventions is aligned with other global health priorities in relation to primary health care (PHC) and universal health coverage (UHC).

Despite this improvement in defining the problem of NCDs, there has been a slow progress in the uptake and implementation of reforms, especially in LMICs. Several countries with substantial burdens of NCDs lag behind in their health system and policy responses to NCDs. The implementation of policy changes and health system reforms remains a tough journey in many settings due to power and political factors, which remain underexplored [4]. This limited attention to policy making dynamics as affected by bargaining powers of multiple stakeholders with different capacities and interests in shaping policies and reforms [4, 5], contributes to the limited knowledge on how to overcome inequities within health systems in regard to NCDs, as well as the underlying factors of the limited uptake of public policies to protect communities from increasing risk of NCDs. Therefore, the political economy analysis of NCD prevention and control becomes essential to better understand the challenges and opportunities of change in local settings i.e., the uptake of adequate ‘technical’ solutions for NCDs [6].

Such analysis is more crucial in settings with complex layers of fragility, such as the middle-income country of Lebanon. Lebanon faces substantive challenges within and beyond the health system, including systemic governance challenges. Lebanon’s health profile is already dominated by NCDs which account for more than 90% of mortality [7]. Mortality and morbidity from cardiovascular diseases, diabetes, chronic respiratory diseases, and cancer increased by at least 30% between 2007 and 2017. Same trends are described for risk factors, with a 50% increase in high body mass index, 40% in tobacco use, and at least 30% in metabolic changes [8, 9]. These figures suggest that Lebanon would not achieve the NCD-related global targets aiming at halting the increase in diabetes and obesity and decreasing the prevalence of risk factors by 2025 [2]. Local research also reveals gaps in screening and early detection of NCDs as well as inequities by coverage schemes and socio-demographic indicators [10, 11]. Moreover, NCD diagnosis and management are not always optimal, with evidence of gaps in hypertension and diabetes control at national levels [12, 13], as well as in the diagnosis and treatment of chronic respiratory diseases [14, 15]. Vulnerable groups, such as Syrian and Palestinian refugees experience more challenges in relation to NCD care [16–19], due to issues of integration of refugees within the national health system [20].

The aim of this article is to conduct a political economy analysis of NCD prevention and control in Lebanon. The specific objectives are: (1) to examine the multifaceted structural features of Lebanon, encompassing political and economic characteristics, which exert a significant influence on the formulation and execution of policies, particularly with regard to the capacity to initiate reforms; (2) to explore the NCD prevention policy landscape and the health system’s capabilities in effectively controlling these conditions; and (3) to identify the power dynamics among various stakeholders and their respective positions towards enabling or inhibiting change and reforms. By addressing these objectives, this research aims to provide a comprehensive understanding of the challenges and opportunities inherent in Lebanon’s policymaking landscape for NCD prevention and control.

## Methods

### Analytical tool and study design

This paper followed a single-case study research design [21], based on a literature review and stakeholder mapping. The units of analysis of the case study emerged from the analytical framework of problem-driven political economy analysis by Fritz and Levy [22], and included: contextual characteristics like the political system (“structural factors” in the framework); policies and health-system decisions and reforms (“institutions”); individuals and organizations (“stakeholder interests, constellations and power”).

The analytical framework suggests defining a problem and then exploring the structural features of the context as well as the institutional and governance arrangements affecting the problem. The analysis also includes the identification of concerned stakeholders along with their motivations, incentives and positions vis-a-vis potential change and their different power levels.

### Context and target population

Lebanon is a low-middle-income country in the Middle East with a population of 4.5 million nationals and about 0.5 million Palestinian refugees as well as 1.5 million Syrian refugees. We acknowledge that the specific situation of Palestinian refugees (including separate health service provision by UNRWA, the UN Relief and Work Agency for Palestine Refugees in the near east) merits its own specific approach and body of work that can complement this research and analysis, which specifically for NCD service delivery, does not cover this population group.

### Data collection and analysis

A literature review was initially conducted between July and September 2018 for the preparation of an outline draft and then regularly updated until the write-up of the final draft of the study results in June 2019. Peer-reviewed publications were retrieved via Pubmed/Medline and Google Scholar. The search process used keywords and MESH terms describing NCDs and their risk factors as well as each unit of analysis (e.g. policies, health system) and their sub-units (e.g. primary care). Examples of the used concepts and terms are included in Additional file 1. Boolean operators and search limitations (e.g. search terms in titles / abstracts only) were used to increase the specificity of the search whenever needed. Research papers using both quantitative and qualitative methods were reviewed. Other search strategies included: searching the reference lists of included papers; and author searching. Beyond the academic literature, data was retrieved from the grey literature such as the websites of governmental institutions (Ministry of Public Health, Ministry of Finance, Central Administration of Statistics etc.), UN agencies (WHO, UNDP Lebanon etc.) and news agencies (BBC, AlJazeera English etc.). Retrieved documents were in English, Arabic and French and no publication date restriction was used. A total of 94 peer-reviewed articles and grey literature publications were reviewed.

Data extraction and analysis followed case study research principles and techniques as described by Yin (2018) [21]. For instance, data extraction included the creation of a “case study database” including narrative summaries of key findings from the included publications. This technique aimed to provide track evidence for the researchers’ interpretations and conclusions in the case study. The overall analytical approach was based on organizing the study results according to the selected framework instead of following theoretical propositions (in a deductive approach) or “working the data from the ground up" (in an inductive approach), while examining different plausible explanations of emerging findings and identifying theoretical underpinnings in the overall discussion of the study findings [21]. This approach was repeated through three progressive stages by the research team, being further refined at each stage. The stakeholder mapping was conducted using a template from an online resource for health systems and policy analysis [23].

## Results

### Structural factors

#### Political system

Lebanon has a parliamentary democracy with sectarian division of high offices, cabinet ministers and parliamentarian seats. The Economist Intelligence Unit in its 2017 report on democracy qualified Lebanon’s political system as a hybrid regime mainly due to gaps in the electoral process, civil liberties, government functioning and the political culture [24].

Lebanon’s history is characterized by continuous political instability with religious power-sharing. The instability started with a civil war between 1975 and 1990 and continued with the burden of both Israel’s occupation of Lebanon’s southern region until 2000 and its military attacks until 2006, as well as the Syrian occupation of Lebanon until 2005. In recent years, Lebanon experienced continuous disruption of presidential and parliamentarian elections and several political crises, including tensions between political parties that almost culminated in another civil war [25, 26]. Some drivers identified for these internal crises include the geopolitical position and the inability to introduce reforms in the political system towards a full secular democracy [27].

In terms of governance features, Lebanon currently shows gaps in government effectiveness, rule of law and control of corruption, compared to similar upper middle-income countries (UMIC) [28]. This situation has affected social welfare in the country. In fact, research on access to welfare and social benefits in Lebanon showed that sectarian actors control the state’s resources and the welfare regime via non-state mediators [29].

#### Economic context

Despite its label as a free and liberal economy, Lebanon’s economic sector serves the interests of a small network of families with major shares in the banking and commercial sectors which account for the majority of the Gross Domestic Product (GDP) [29]. GDP growth was badly affected in recent years due the continuous political turmoil and the impact of the war in Syria on major sectors in Lebanon such trade and tourism [30]. Moreover, Lebanon has one of the highest public debt-to-GDP ratios in the world [31]. Additionally, corruption and inefficiency contribute to limiting the government’s ability to invest in infrastructure and the provision of public services, especially with the increasing demand from both refugee and host communities [32]. In their analysis of the government public spending after the civil war, Salti and Chaaban identified irrational spending decisions not matching needs and rarely attaining their objectives [33]. The consequences for communities are increased poverty and unemployment rates as well as widened wealth inequities. Poverty rate stands at 27%, with striking regional differences and unemployment rate was around 11.4% in 2018 with age and gender inequities favouring men and adults compared to women and youth [34]. Wealth distribution is highly unequal in Lebanon with a global rank of 129 out of 141 in terms of income equity using the Gini coefficient [35]. Syrian refugees in Lebanon, in particular, experience high poverty levels and tough employment policies, with a situation expected to worsen given the increasing humanitarian funding gap in recent years [36].

#### Demographic factors

Population ageing has put the country at the top of Arab countries with the highest proportions of older adults. About 12% of the population are of age 60 years or older [37]. Life expectancy at birth reached 80 years in 2012, compared to 68 years in the corresponding WHO region and 74 years in the corresponding World Bank income group [38]. In fact, Lebanon witnessed a rapid demographic transition within the last three decades of last century, with fast declines in fertility and mortality [39]. In 2017, the percentage of people living in urban areas of Lebanon was 88.4% compared to 42.3% in 1960. Greater Beirut including the capital city of Beirut and surrounding areas is the residence for almost one-third of the whole population of Lebanon, including refugees [40].

#### International or exogenous factors

Lebanon presents no exception to the impact of the well-established association between global trade and investment policies and NCD drivers in LMICs [41], especially with its liberal free-market economy. Evidence from local research proved the interference of transnational tobacco industry for decades in public policy making in order to avoid anti-marketing policies and secure market access in Lebanon and the Middle East region [42]. Other unhealthy products like alcohol and sugary beverages have high levels of per-capita consumption compared to the region [43, 44]. Finally, Lebanon has witnessed changes in nutritional characteristics towards high-fat hyper-caloric diets; this is, on one hand due to the increased availability and affordability of unhealthy food in the market, and, on the other, to the historically import-dependent economy that may have deterred local agricultural development and the present-day availability of varied and healthy locally-sourced food alternatives [45].

### Institutional level: substantial prevention policy gap, fragmented health system and unmet NCD care needs

#### NCD risk reduction policy landscape in Lebanon

The policy responses targeting NCD risk factors in Lebanon can be described as very limited. Lebanon signed the WHO Framework Convention on Tobacco Control (FCTC) treaty in 2004 and ratified it 1 year after. However, it took 6 years with intermittent discussions around law proposals to pass the Law No.174 “Tobacco Control and Regulation of Tobacco Products’ Manufacturing, Packaging and Advertising” in September 2011. Although the law tackled several aspects of tobacco control, strict compliance to the indoor smoking ban in public places lasted for about 1 year [46], and it is currently considered moderate (score: 3/10) [47]. Bans on advertising, promotion and sponsorship are moderately implemented as well (score: 6/10). Anti-tobacco health education initiatives are limited: health warnings exist only on 40% of all display areas of tobacco products and do not include photographs or graphics; and no anti-tobacco mass media campaigns have been implemented since 2013. As for fiscal policies, the percentage of taxation is about 45% of the retail price [47]. This level is below the WHO recommended threshold of 70% which is implemented in several countries in the Eastern Mediterranean region [47].

Gaps in the alcohol- and diet-related policy landscape are even more serious in Lebanon. We identified only the following alcohol-related policies: (1) the traffic law that stipulates a limit for the blood alcohol concentration while driving [48], and (2) the negligible taxation on alcohol products ranging between 60 – 200 Lebanese pounds/litre (~0.04 – 0.13 USD/litre) [49]. Moreover, there are no substantial regulations that limit the physical availability and affordability of alcohol in Lebanon nor its advertising and marketing [50]. For diet policy options, the country experiences a real vacuum of policy-making despite a few initiatives promoted by academia to reduce salt consumption through the reformulation of food products [51]. Evidence-based policy options such as labelling policies and taxation on sugar-sweetened beverages, as well as national public education initiatives, are absent [7].

Data on physical activity policy response in Lebanon is lacking. According to the WHO NCD Progress Monitor [52], Lebanon has not implemented any free national mass participation events for physical activity or national public education and awareness campaigns in recent years. In relation with structural factors especially in urban settings such as Beirut, there is a significant negligence of social elements of urban planning, resulting in bad-quality public transport system and construction plans with no focus on walkability, cycling and/or the preservation of green spaces [52]. Urban residents rely therefore on private cars for land transportation [53]. Health consequences of urban residence are identified by few research studies. Sibai, Costantin et al. reported a positive association between urban residence and physical inactivity among Lebanese adults [54]. Moreover, a significant association between poor neighbourhood and chronic illness in an underserved urban community in GB was also identified [55].

In terms of NCD strategies and action plans, the MOPH developed a national NCD prevention and control plan (NCD-PCP) in 2016 with the support of the WHO country office and after consultation meetings with other governmental and non-governmental stakeholders. Although the plan has comprehensive and ambitious strategic directions for NCD prevention policies, the MOPH did not achieve the first-year objective (i.e. establish “National NCD Task Force” with focal points within relevant government bodies). Moreover, a specific budget and qualified human resources to push the NCD policy agenda within the MOPH have not been ensured. Therefore, Lebanon was unable to achieve the outcomes of its own national plan, the indicators of the WHO regional framework for action on NCD as well as the WHO global NCD voluntary targets for 2025 [2, 56].

#### Lebanon’s fragmented health system: coverage and service delivery status

Descriptive analysis of the health system in Lebanon is presented in this section to compare the status of health coverage and service delivery with both UHC and PHC frameworks given their relevance in reforming the system to deliver better NCD care. This framing of the analysis is supported by the dominance of Lebanon’s health profile by NCDs and intention to move away from a vertical disease-focused to a health system strengthening approach.

##### Health coverage challenges in Lebanon

Health coverage in Lebanon is characterized by the multitude of coverage schemes, each with their own policies on cost-sharing and what services are to be provided. Six public funds operating under the tutelage of different government bodies cover almost 43% of the Lebanese population [57]. The National Social Security Fund (NSSF), an autonomous public organization under the supervision of the Ministry of Labour, is the largest one that mainly covers employees of the formal private sector. Based essentially on financial contributions from both the employers and the employees, the NSSF aims to cover 85% of outpatient care fees in private clinics and 90% of hospital care – including NCDs. The remaining funds are the Civil Servants’ Cooperative (CSC) and the health funds of 4 military service bodies, each with a separate administration and reporting line within the government but all mainly funded by general taxation. Non-adherents to any of these public funds who cannot afford private insurance plans are estimated at 45% of the Lebanese population. Those are eligible to be covered by the Ministry of Public Health (MOPH) that acts as a “payer of last resort” and covers 85% to 95% of hospital care fees and provides expensive drugs for catastrophic illness like cancer. The MOPH does not cover consultation fees in private clinics or the purchase of drugs from private pharmacies. However, it delivers vaccines and essential drugs to a network of health centres that are mainly run by civil society and municipalities to serve poor communities [57].

Syrian refugees are not eligible to join any public scheme nor to be covered by the MOPH. However, the UN Refugee Agency (UNHCR) covers outpatient care through Lebanon’s network of health centres, and hospital care in Lebanese hospitals only for obstetric and life-threatening conditions, with a 25% co-payment by beneficiaries. Several conditions remain uncovered including the very expensive cases of cancer and renal dialysis [58].

Because of this fragmented and limited coverage, there is a high financial burden on households. In fact, households contribute to a major part of the health bill either by premia (17%) to public funds and private insurance or by out-of-pocket (OOP) payments (37%) [57]. Health expenditures, estimated at 5.9 billion USD in 2014, are expected to increase to 9.6 billion USD in 2040 and the percentage of OOP is not expected to decrease [59]. Beyond this national figure, spending on healthcare varies by socio-economic status. A micro-economic analysis of health equity in Lebanon showed lower rates of insurance coverage among the poorest, leading to higher proportion of expenditures on health [60], i.e. exacerbated inequities in health coverage. Moreover, the NSSF has longstanding financial and governance challenges including liquidity issues due to increasing amount of money owed by the Lebanese government to the NSSF [61].

##### Service delivery distortion toward specialist-led and commercialized care

During and after the civil war, Lebanon’s health sector has evolved around private hospitals and clinics delivering sub-specialized care [62]. These entities are the main provider of health services and mainly financed by public funds. Private hospitals deliver more than 80% of secondary and tertiary care in Lebanon [63]. Although there have been achievements in regulating the private sector (see below), the oversupply of hospital services and technologies along with fee-for-service reimbursement policies has led to over-demand and inefficiency in public fund allocation [57]. For instance, Sibai et al. [64] reported a high level of inappropriate use of coronary angiography procedures, which correlated with the per-capita density of cardiac catherization laboratories. Overall, only 55% of the procedures were appropriate according to clinical guidelines.

Outpatient care in Lebanon is also affected by the overall distortion of the system towards commercialized private practice. Lebanon’s physicians are mostly specialists working in private practices and getting paid out of pocket from beneficiaries who might be eligible for reimbursement from public funds (excluding the MOPH) and private insurance. Private practitioners are the most common entry point to the health system, especially for people with health coverage. Uninsured Lebanese and non-Lebanese who cannot afford private consultation fees access NGO- and municipality-owned centres. The ministry supports these centres with medicines and equipment as well training and capacity building, while in return facilities commit to abide by the MOPH consultation fees and regulations [65]. In summary, NCD care is mainly provided in private hospitals and clinics and twisted towards highly specialized approaches which might not meet the primary needs of beneficiaries.

### Stakeholder analysis

In this section, we present the stakeholder analysis which unpacks the power and positions of key actors who were involved in the NCD prevention policymaking and reforms of care provision in Lebanon.

#### NCD prevention

**Fig. 1:**
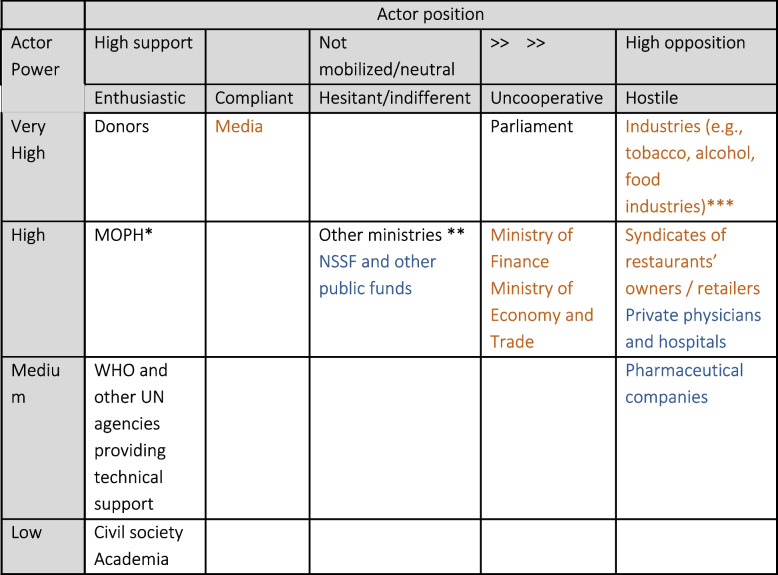
Stakeholder mapping regarding NCD prevention policy change and care model reforms in Lebanon^#^. * Depending on the availability of funding and qualified human resources. ** e.g., the Ministry of Tourism in the case of tobacco control. ***Food industry could be compliant depending on policy options. Colour coding: black = common across policies; blue = service delivery reforms; orange = NCD prevention policies. # This mapping is based on described policy processes in the literature and intend to be informative for next policy making attempts. Actors can change their positions depending on the specific policy being explored and depending on the interaction between themselves

Regarding NCD prevention, we drew on the experiences of Lebanon with tobacco control [46, 66], and nutrition-related policymaking [51]. The analysis showed that NCD prevention policymaking has been under the influence of stakeholders with contrasting interests, powers and networking strategies. Figure 1 shows the distribution of these stakeholders based on their power and interests to support NCD prevention policymaking. However, this figure is not a prescriptive reality as future policymaking processes might have different dynamics but gives policy advocates a basic understanding of the allocations of different potential actors.

Both cases suggested that the MOPH is clearly in the centre of the policymaking network but usually lacks the needed capacities and resources. The civil society and academic institutions were identified as trusted partners to the MOPH. Moreover, international donors (e.g. and technical UN agencies like WHO) showed consistent support for NCD prevention policy-making efforts, but with different power levels: funding agencies have been more powerful than UN agencies which provide technical support only. The main example was the financial support from Bloomberg foundation (Bloomberg Global Initiative – GBI) to the National Tobacco Control Program (NTCP) in 2010. which triggered the opening of a window of opportunity and led to the creation of Tobacco Law 174. The position of industries varied depending on the type of policy experience: although some food industries (e.g. bakeries) showed an intention to collaborate with MOPH and/or its allies, the tobacco industry was at the highest levels of opposition and power possession and influenced many the position of other stakeholders (e.g. Ministry of Tourism and parliamentarians). Finally, governmental agencies (e.g. Ministry of Economy, Ministry of Interior, and Ministry of Industry) and the Parliament usually start with a neutral or uncooperative position and move into either supportive or opposed status depending on the influence of policy clusters. Media, which influenced the public opinion and indirectly politicians to prioritize public health concerns over the interests of the private sector, can be considered as a compliant and very powerful stakeholder if alliances with NCD policy proponents are established [46, 66].

In addition to external funding, other enablers of policy change could be extracted: a supportive political environment (e.g. the relative stability in Lebanon during 2009–11 which followed the 2008–09 crisis), the availability of committed technocrats, and the ability to create strong coalitions including with media to start evidence-based discussions/campaigns and influence the public opinion and the policymaking process. Moreover, previous experiences identified a potential for policy advocates to overcome the loopholes in the political system to secure better policy outcomes in the future. However, the lack of a dedicated governmental unit for NCD prevention, and therefore the sustainability of financial and human resources was identified as barrier to effective policy change.

#### NCD care delivery

Lebanon’s health sector has experienced over the last two decades several reforms at the levels of both hospital and outpatient care delivery as well as health financing, due to the engagement of key supportive stakeholders: mainly the MOPH and its allies in the civil society, academia, and international technical and funding agencies (e.g., World Bank and WHO) (see Fig. 1).

During the post-civil war period (1991 – onwards), the MOPH has established an alliance with the civil society, academia and international organizations to: (1) generate evidence on burden of disease and conduct national health accounts, (2) to assess the situation of health centres and hospital contracting arrangements [62], and (3) to introduce policies/regulations addressing the fragmentation of the system and regulating the private sector. The MOPH led several projects to invest in the public hospital infrastructure with the support of the World Bank and donors [62], and supported several reforms that changed the administration of public hospitals and the contracting mechanisms for all hospitals, such as providing more autonomy to public hospitals, re-visiting of the reimbursement policy of inpatient services (moving away from free-for-service) and the pricing structure of lab and imaging testing and other supplies [57, 67].

The Ministry of Public Health has also been the key stakeholder to address health inequities in relation to primary care in Lebanon. For that, it has invested since the early 1990s in strengthening the PHC system. The PHC network has rapidly grown from only 28 primary health care centres to 86 centres in 2005 and currently more than 220 centres [65]. It has become the base for several national vertical initiatives and programs implemented by the MOPH in collaboration with its local and international partners. In relation to NCD prevention and management, a Chronic Medication Program was established in 1993 to provide chronic medicines at a nominal price to vulnerable populations. It served around 150,000 Lebanese beneficiaries and 15,000 Syrian refugees in 2018 [65]. The program is led by a local NGO, with experience in delivering medicines during emergencies, which has been managing an NCD-related programme for the MOPH. The MOPH has also contributed to the structural improvement of health facilities by providing equipment mainly through donations, as the allocated general budget for PHC is estimated to be less than 10% of the MOPH budget [68]. With regards to cardiovascular disease (CVD), the MOPH, with the technical support of WHO, started an initiative to detect and treat CVD metabolic risk factors using the WHO-ISH CVD risk stratification method [69]. Moreover, clinical guidelines were developed in collaboration with academia and the Lebanese Society of Family Medicine and with the financial support of the European Union (EU), which continued to support PHC strengthening projects such as the Emergency Primary Healthcare Restoration Project (EPHRP) [70]. Beyond the scope of the PHC network, the MOPH has contributed to reducing OOP expenditures through continuous work on decreasing the prices of pharmaceuticals and the promotion of the use of generics [71].

Despite achievements in strengthening the delivery of PHC services by NGO-run facilities, there has been a weakness in regulating the private outpatient sector, which is led by a powerful group of private physicians with close relationships with private hospitals and the pharmaceutical industry. This power imbalance between the private sector and the government puts more roadblocks in the progress towards universal health coverage, especially within the challenging political environment as previously described. Previous attempts to expand the coverage of the NSSF health fund also failed mainly because of the political environment and governance issues [72].

Currently, the health system still lacks key elements of a strong PHC model, including the absence of a clear close-to-communities entry point to the system (outside the premises of hospital outpatient clinics) and a clear coordination or referral mechanism between different levels of care. Moreover, the absence of a clear strategy for health financing impedes the establishment of one sustainable universal health coverage system delivering efficient and high-quality care. Finally, a clear approach for community engagement in order to build trust in primary care is not available.

## Discussion and conclusions

**Fig. 2:**
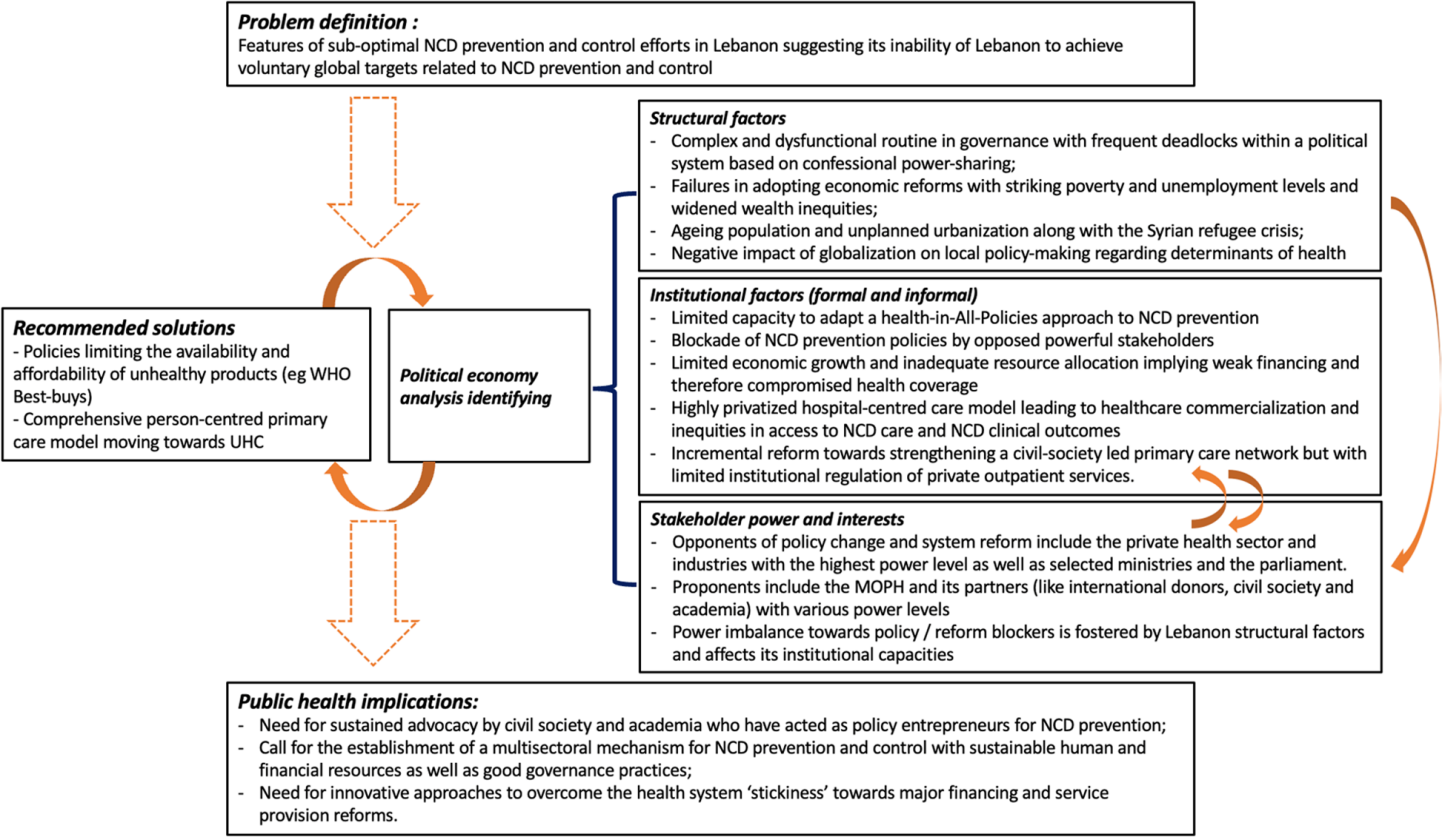
Political economy of NCD prevention and control in Lebanon (adapted from Fritz and Levy, 2014) [22]

This political economy analysis explored the interaction between Lebanon’s structural factors and the stakeholders’ positions towards shaping institutional changes to improve NCD prevention and control. Lebanon’s political instability and fragile governance negatively affect its capacity to adapt a Health-in-All-Policies approach to NCD prevention as well as major health system reforms towards a more equitable care model, by allowing powerful opposed stakeholders to block or delay any policy-making that is against their interests. Lebanon’s economic hardship and reform failures imply weak financing and inadequate resource allocation, resulting in compromised health coverage (with high OOP expenditures as a major symptom) and health inequities. NCD care provision is twisted by the powerful private sector towards a hospital-centred model with aspects of commercialisation of care. Stakeholders like the MOPH, UN agencies, and NGOs have been pushing towards changing the care model towards comprehensive person-centred primary care. An incremental reform to strengthen a network of primary care centres, support them with health technologies and improve the quality of PHC services has been identified. Nevertheless, outpatient services that are covered by other public funds – serving at least half of the population – remained specialist-led without much institutional regulation. Figure 2 summarizes all these findings.

### Theoretical reflections on policy change and reform dynamics

Theories from the policy literature, such as ‘incrementalism’ and ‘punctuated equilibrium’ [73], can be used to explain the dynamics of policy change and system reforms identified in this case study.

‘Incrementalism’ described policy-making processes that are based on consecutive incremental steps moving from an initial policy status into a nearby alternative that secures an agreement between different stakeholders. This theory explained these processes using two relevant concepts: institutional ‘stickiness’, that means the resistance of institutions to change because of political deadlocks by interest groups; and ‘path dependency’, that means the limited availability of policy options via a closed path for policymaking based on previous experiences. In our case study, this theory applies to the ‘stickiness’ of the NCD care provision model and the inability of MOPH and its allies to go beyond an incremental progress in order to impose a radical reform. Even though some current initiatives could have fed strategically into the model of comprehensive person-centred primary care, their contribution could not overcome the major structural gaps of the system.

The theory of ‘punctuated equilibrium’ argues that powerful interest groups or governing elites monopolize policymaking in a long-standing equilibrium by framing problems and their solutions according to their ideas and interests. Substantial changes can then occur when contextual shocks disturb this status-quo of policymaking. The ‘punctuated equilibrium’ theory applies to Lebanon’s experience with NCD prevention policymaking because our findings suggest that policy monopolies in Lebanon retain legislative and executive policymaking power regarding NCD determinants (e.g. tobacco control) in a locked equilibrium, with potential opportunity windows relying on exogenous push factors (like international treaties and funding). To provide further explanation to this phenomenon, especially in terms of the additional factors (along with the shocks) needed to enable change, we report the findings of a scoping review that investigated the design and implementation of health taxes on NCD risk factors in LMIC. This review identified additional enablers such local evidence, policy championing and inter-ministerial support [74]. The study also revealed obstacles like negative framing and retaliation by industry, vested interests, and governmental policy disjuncture [74].

### Advocacy and problem framing

The political economy of NCD reveals powerful corporations and interests. Public health policymaking can fall down when the power balance is weak. While this is the case in many countries – especially those with limited governance strength –, public health practice and policy theories do indicate that lobbying and advocacy by groups of actors can trigger change. Growing literature discusses the role of advocacy alliances and ‘policy entrepreneurs’ or ‘policy brokers’ to influence public policy by seizing the change opportunities [75]. In Lebanon, civil society, including academia and NGOs, has shown a potential to generate policy entrepreneurs who can build alliances with bureaucrats and media and influence the national dialogue in favour of NCD policy reform, as shown in many units of analysis of this case study.

Problem framing was identified as a key determinant of the success (or not) of the policy process. Unwin et al. [76] noted the importance of problem framing to encourage NCD policy development in Barbados. Moreover, increased awareness of the NCD burden among the public through media coverage has contributed to government commitment in Barbados. Although public awareness of secondary prevention of NCDs, such as breast cancer screening, is strong and influences policymakers’ decision to support it [11], more efforts are needed to increase population knowledge about NCD primary prevention (especially tobacco and obesity control) and the distortion of the health care system and frame those topics as policy problems needing a high-level legislative response. The emergence of those ideas is essential to overcome reform opponents. Experiences from high-income settings validate this recommendation of good problem framing and its impact on getting the public on side for NCD public policy action.

### Monitoring policy implementation and good governance

The translation of desired change into new legislations or regulations should not be the final outcome of policy advocacy. Higher outcomes include implementing new policies as proposed, attaining their intended consequences, and translating them into new societal norms. The lesson from the 2010–11 tobacco control experience is that it is essential for policy promoters to understand the power of opposed stakeholders in reversing government commitment to public health even after policy creation. Future policy initiatives should incorporate clear monitoring and evaluation indicators. Moreover, reform proponents need to monitor the public-private partnership in policy creation and implementation. The relationship between the government and some private organizations like food industries and pharmaceutical companies, unlike tobacco and alcohol industries, is essential but might induce conflict of interests [77]. Overall, any collaboration between stakeholders should be embedded in good governance practices [78], including multisectoral collaboration, community engagement in decision making and strong monitoring systems and accountability mechanisms [79].

### Strengths and limitations

This is among the first studies to explore the political economy of NCD prevention and control in Lebanon. The analytical tool and the theoretical reflections on key findings provided an opportunity to understand this complex problem by combining different layers of analysis and to extract relevant public health practice implications. The case study research design is usually criticized for not being rigorous enough and providing ungeneralizable findings. Our study, however, used a detailed analytical framework in order to achieve a structured presentation of findings. In terms of generalizability, this study, as for many other health policy studies, does not aim primarily to generalize evidence but rather to understand phenomena in local settings and whenever possible to generalize the use of frameworks and theories to add to the body of literature on the topic.

Future research on the political economy of health in Lebanon, should extend beyond the boundaries of our article and use various data collection techniques (e.g., qualitative interviews, participatory methodologies, archival and ethnographic fieldwork) to further unpack the complexity of this topic. This article along with a recent report on the political economy of health in Lebanon that examined the intricacies of decision-making, governance and health financing in Lebanon including during major events such as the current economic crisis that started late 2019 [80], serve as the foundation for a substantial body of needed forthcoming work. Nevertheless, both publications generated data that underscore the importance of strengthening the Lebanese health system, moving towards universal health coverage and primary care-oriented model of care to ensure equitable health services and protect communities, especially in times of limited resources.

### Supplementary Information

**Additional file 1.** Examples of concepts and search terms used in identifying literature on NCD prevention and control in Lebanon.

## Data Availability

All data generated or analysed during this study are included in this published article.
