# Supplementary material for: Political Economy of Non-Communicable Disease (NCD) prevention and control in Lebanon: identifying challenges and opportunities for policy change and care provision reforms
Source: BMC Public Health. 2023 Dec 18;23:2526. doi: 10.1186/s12889-023-17357-1 (PMC10726601; doi:10.1186/s12889-023-17357-1)
Supplement: Supplementary file 1 — Additional file 1. Examples of concepts and search terms used in identifying literature on NCD prevention and control in Lebanon. [file 12889_2023_17357_MOESM1_ESM.docx]

Additional file 1. Examples of concepts and search terms used in identifying literature on NCD prevention and control in Lebanon

| Concept | Examples of used search terms in different databases |
| --- | --- |
| Non-Communicable Diseases and their risk factors | Non-communicable disease* OR chronic disease* OR hypertension OR diabetes OR cardio-vascular disease* OR chronic respiratory disease* OR Cancer OR diet OR physical activity OR smoking OR alcohol |
| Policies | Health* policy OR healthy public policy OR public policy OR best-buy* interventions OR health promotion OR Health in all policies OR intersectoral action OR intersectoral collaboration OR multisectoral action |
| Service provision | Chronic care model OR disease management OR primary care OR primary healthcare OR integrated care OR person-centred* OR continuity of care OR access OR care utilization OR health service* |
| Lebanon | Lebanon OR Lebanese |
